# Supplementary material for: Identification of Cancer Associated Fibroblasts Related Genes Signature to Facilitate Improved Prediction of Prognosis and Responses to Therapy in Patients with Pancreatic Cancer
Source: Int J Mol Sci. 2025 May 19;26(10):4876. doi: 10.3390/ijms26104876 (PMC12112120; doi:10.3390/ijms26104876)
Supplement: Supplementary file 1 [file ijms-26-04876-s001.zip › ijms-3598111-supplementary.pdf]

Identification of Cancer associated fibroblasts related genes signature to facilitate improved prediction of prognosis and responses to therapy in patients with pancreatic cancer

Supplementary Figures

Supplementary figure 1. Overall survival analysis by log-rank. Based on different scoring methods, K-M curves show the pancreatic cancer patients with higher stromal-score related to bad overall survival in TCGA-PAAD.

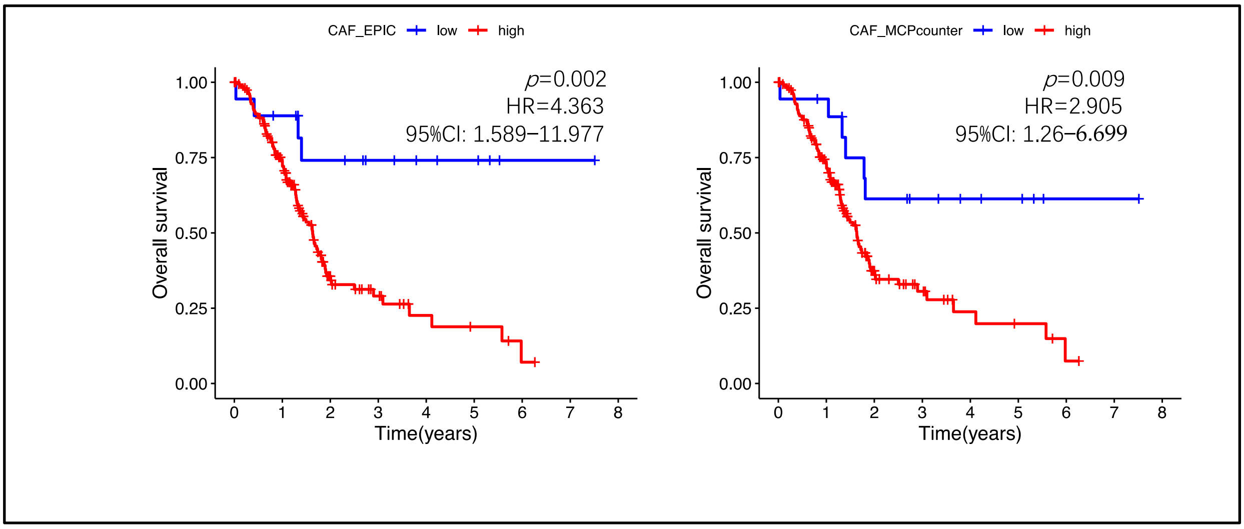

Supplementary figure 2. The topological overlap plot verified the reasonable of our different modules in GSE183795 & GSE78229 (A) and TCGA-PAAD (B)

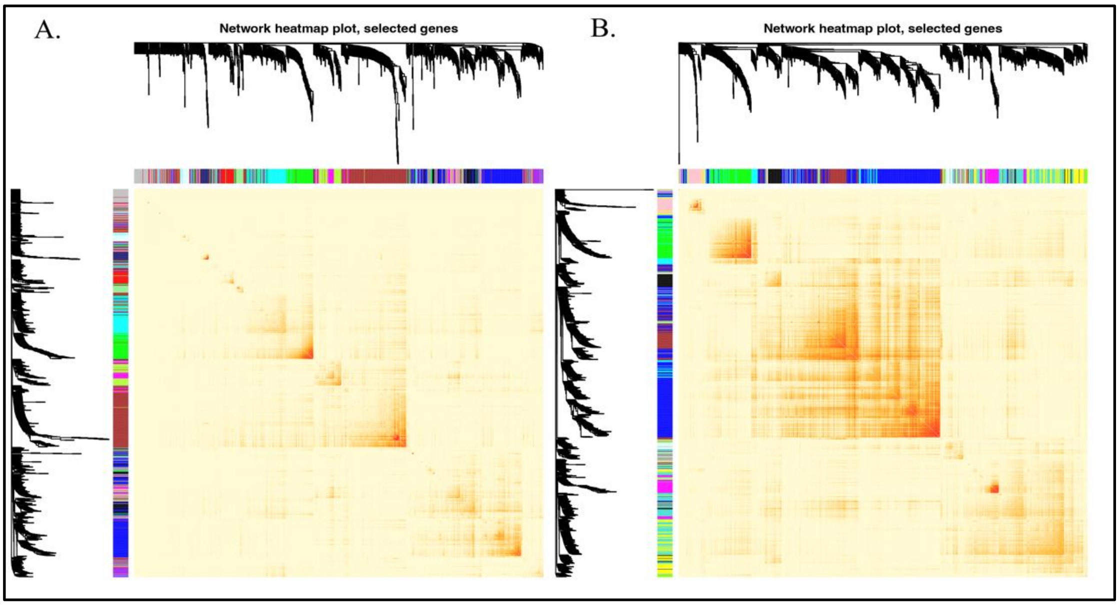

Supplementary figure 3. Clustering dendrograms showing genes with similar expression patterns were clustered into GSE183795 & GSE78229 (A) and TCGA-PAAD (B) co-expression modules.

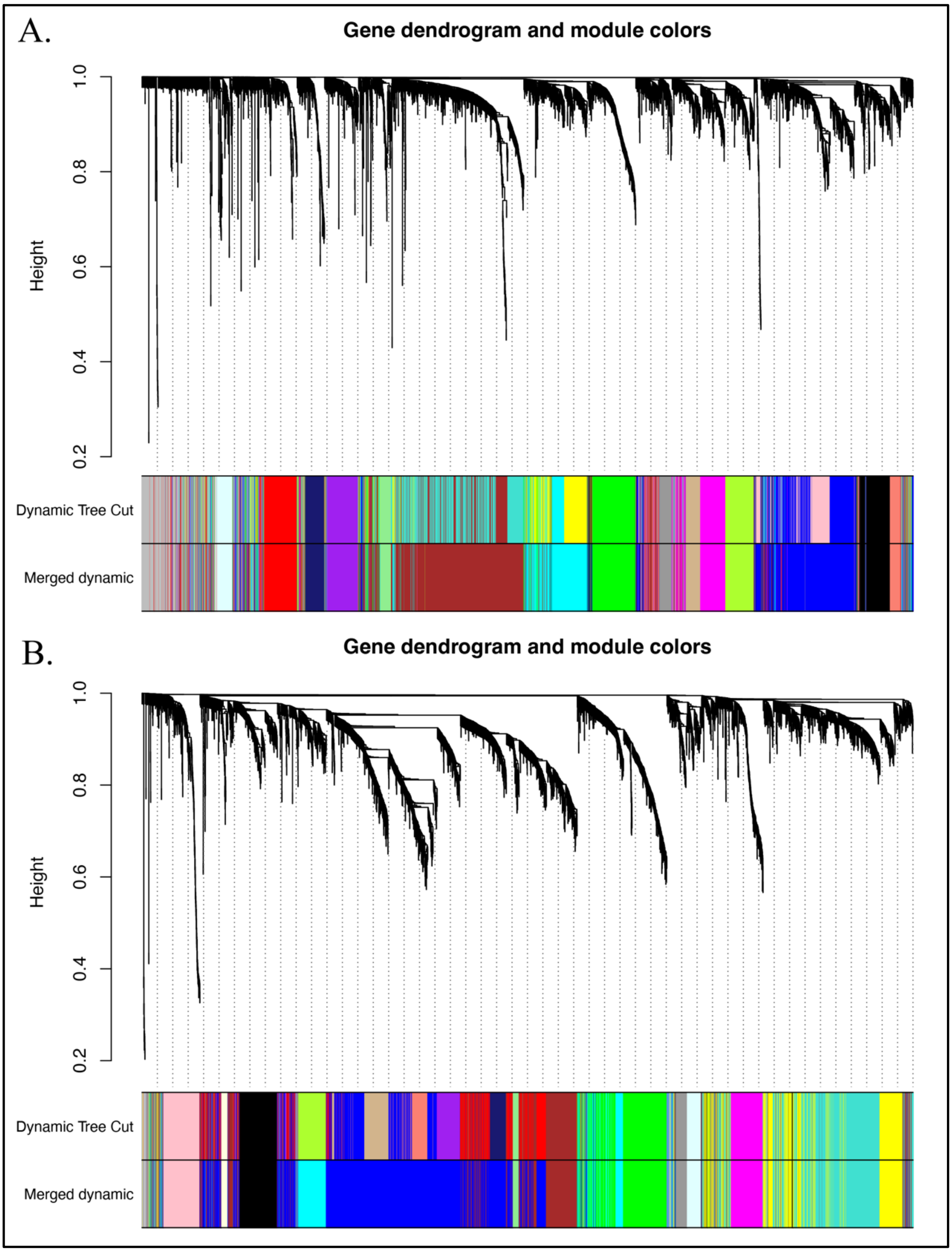

Supplementary figure 4. GO analyses of the enriched 32 genes in the function of biological process (BP), cellular component (CC), and molecular function (MF) terms are shown in the box plot (A) and bubble plot(C). KEGG enrichment of the intersection genes. Showing enriched in cytoskeleton in muscle cells in bar chart (B) and bubble plot (D). Coefficient profiles of least absolute shrinkage and selection operator (LASSO), Cox regression analysis (E), and the adjustment parameter (lambda) (F) were calculated based on the partial likelihood deviance with ten-fold cross-validation.

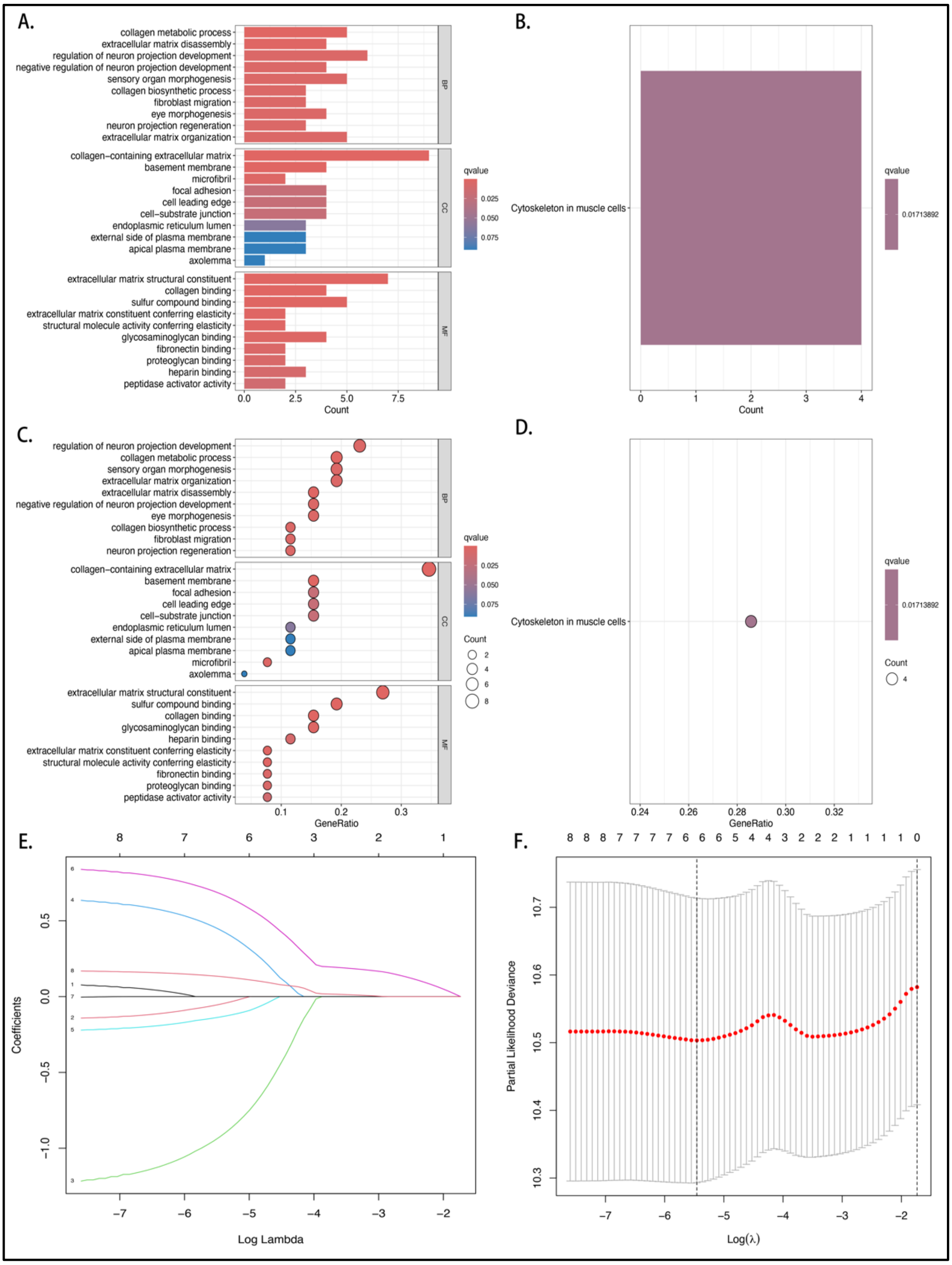

Supplementary figure 5. Tumour Mutation Burden. It shows the difference between the low- and high- CAF infiltration groups (A) and the changes in risk score (B). The difference between the tumour mutation burdens in high- and low- CAF infiltration groups (C) and the changes in risk score (D). Spearman`s correlation analyses revealed that the TMB value was negative related with CAF activations and infiltrations (E).

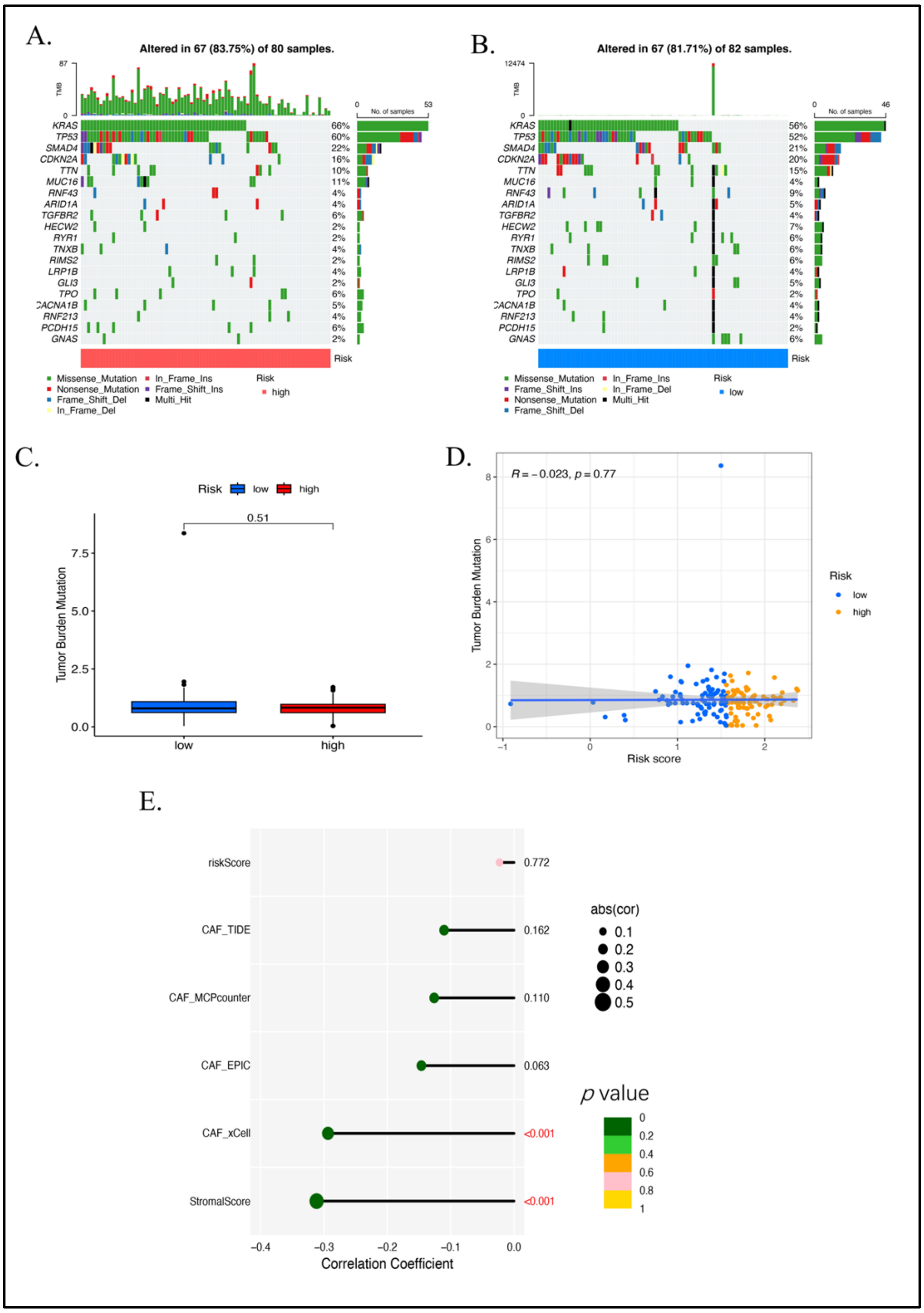

Supplementary figure 6. GSEA and ssGSEA enriched GO biological function in TCGA datasets. (A-B) Gene set enrichment analysis (GSEA) sets between high-CAF risk groups (A) and low risk group (B) in TCGA-PAAD. ssGSEA results showed that the CAF risk score was positively correlated with bone morphogenesis (C), cell fate specification involved in pattern (D), embryonic neurocranium morphogenesis (E), fibroblast growth factor receptor signaling pathway (F) and mesenchyme development (G) in TCGA-PAAD.

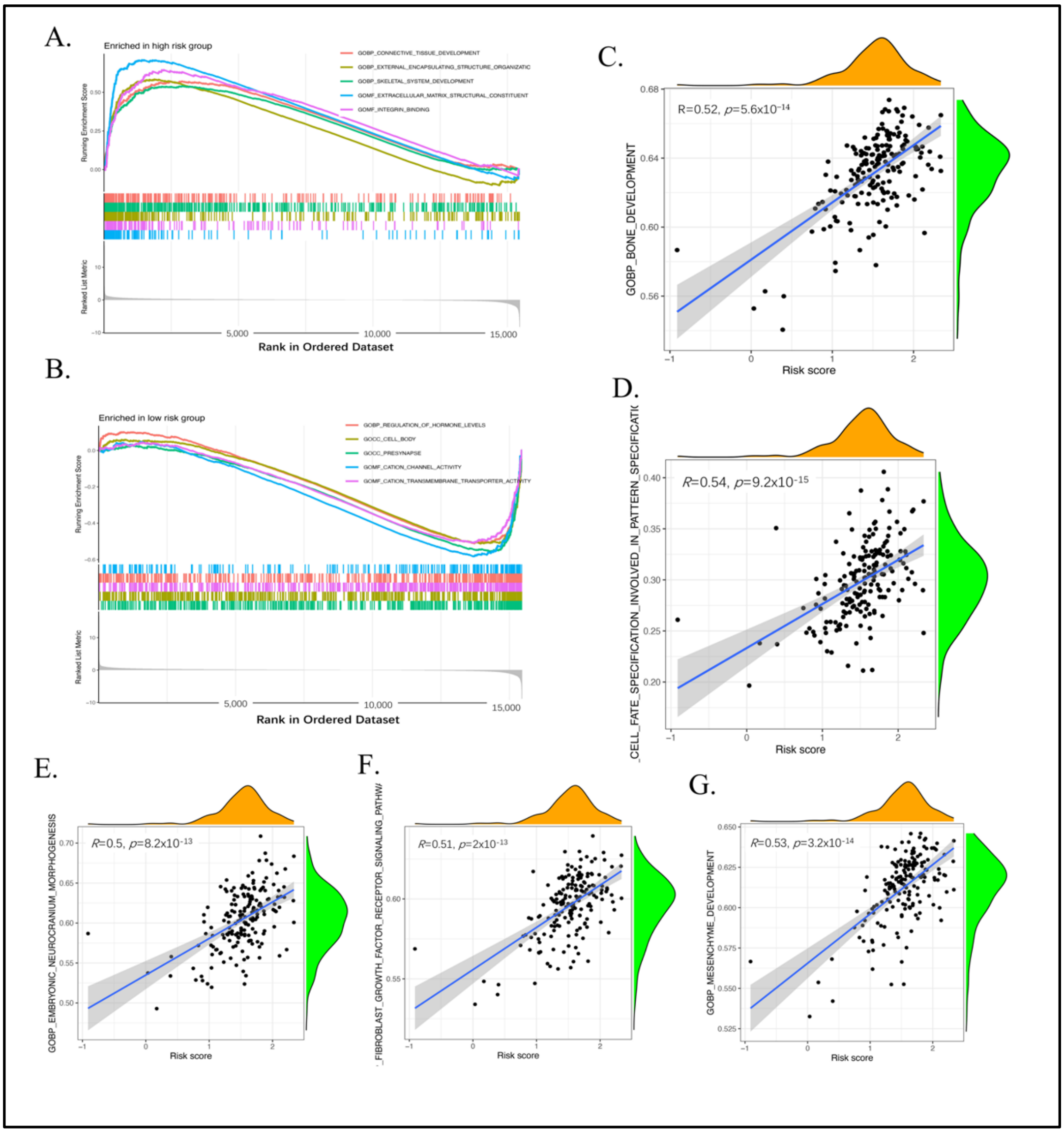

Supplementary figure 7. GSEA enriched KEGG pathway in GSE183795 and GSE78229. GSEA enrichment in high risk group (A) and low risk group (B).

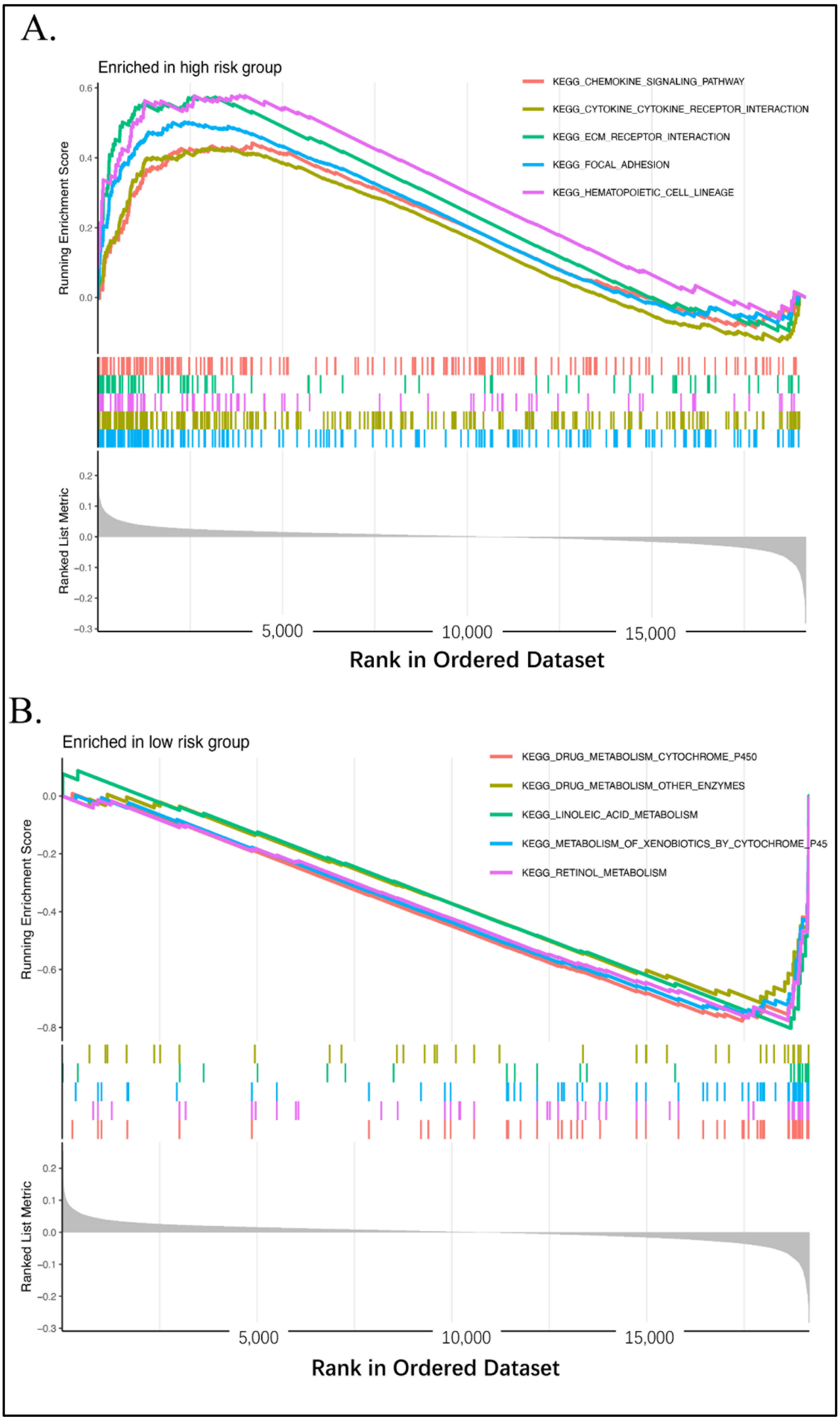

Supplementary figure 8. GSEA enriched KEGG pathway in TCGA-PAAD. GSEA enrichment in high risk group (A) and low risk group (B)

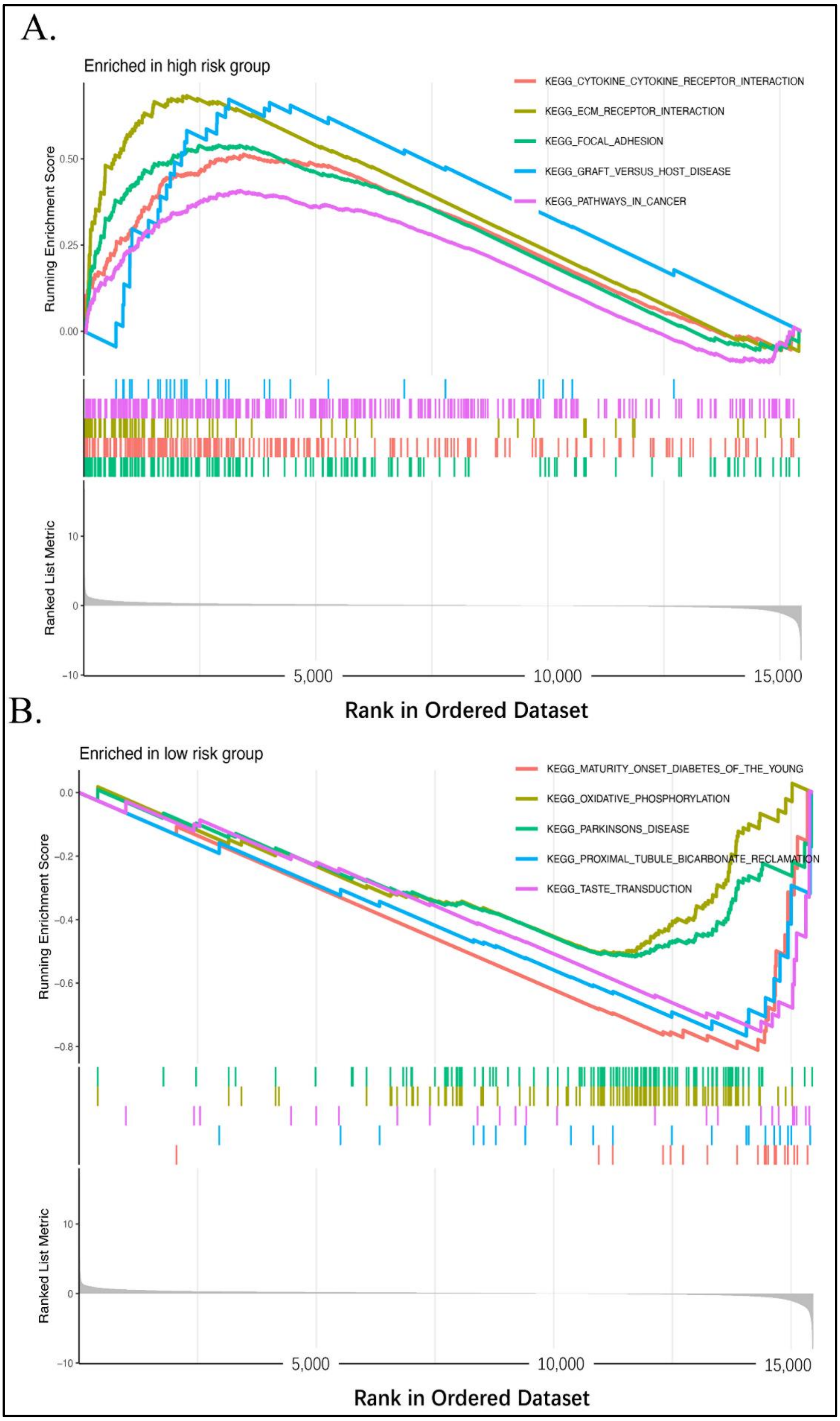
A.
Enriched in high risk group
KEGG_CYTOKINE_CYTOKINE_RECEPTOR_INTERACTION
KEGG_ECM_RECEPTOR_INTERACTION
KEGG_FOCAL_ADHESION
KEGG_GRAFT_VERSUS_HOST_DISEASE
KEGG_PATHWAYS_IN_CANCER
Running Enrichment Score
0.50
0.25
0.00
Ranked List Metric
10
0
−10
5,000
10,000
15,000
Rank in Ordered Dataset
B.
Enriched in low risk group
KEGG_MATURITY_ONSET_DIABETES_OF_THE_YOUNG
KEGG_OXIDATIVE_PHOSPHORYLATION
KEGG_PARKINSONS_DISEASE
KEGG_PROXIMAL_TUBULE_BICARBONATE_RECLAMATION
KEGG_TASTE_TRANSDUCTION
Running Enrichment Score
0.0
−0.2
−0.4
−0.6
−0.8
Ranked List Metric
10
0
−10
5,000
10,000
15,000
Rank in Ordered Dataset
